# Floor-Hugging intervention: findings from an exploratory study of a novel floor-exposure and post-fall contingency program

**DOI:** 10.3389/fresc.2026.1730161

**Published:** 2026-05-29

**Authors:** Shashank Ghai

**Affiliations:** 1 Department of Political, Historical, Religious and Cultural Studies, Karlstad University, Karlstad, Sweden; 2 Centre for Societal Risk Research, Karlstad University, Karlstad, Sweden

**Keywords:** concern of falling, fall management, falls, floor rise training, healthy ageing

## Abstract

**Introduction:**

Falls constitute a significant public health burden across the adult lifespan, affecting both working-age and older populations and compromising functional independence and quality of life through physical and psychological consequences. Current interventions typically address these dimensions in isolation, emphasizing fall avoidance rather than equipping individuals to manage and recover from falls when they occur.

**Methods:**

This exploratory study evaluated the feasibility, acceptability, and safety of the Floor-Hugging Intervention (Floor-HI), a novel multicomponent program that combines controlled exposure to fall-risk environments with systematic training in post-fall recovery skills, including floor-to-standing transitions, fall imagination, and role-playing scenarios. Eight community-dwelling, middle-aged adults (3 males, 5 females; aged 31–55 years) completed a 3-week program. Outcomes including concern of falling, postural control, floor-rise ability, turning ability, and health-related quality of life were assessed at four time points (Weeks 0, 3, 6, and 9).

**Results:**

Recruitment, retention, and adherence rates were 100%, 89%, and 100%, respectively. Significant improvements over time were observed in postural stability (Mini-BESTest, *p* = .011), floor-rise ability (Sitting Rising Test, *p* = .011), and turning capacity (360° Turn Test duration, *p* = .012–.017). Concern of falling (Falls Efficacy Scale-International, *p* = .145) and health-related quality of life (RAND-36, *p* > .05) remained unchanged. Intervention acceptability was high across all domains of the Theoretical Framework of Acceptability questionnaire. Only a small number of mild, transient adverse events were reported, all resolving without medical intervention, and no serious adverse events occurred.

**Discussion:**

Despite being limited by the absence of a control group and a small sample size, this study demonstrates that Floor-HI is feasible, acceptable, and safe in community-dwelling middle-aged adults. These findings provide a foundation for future feasibility and controlled trials to establish efficacy and applicability in populations with elevated fall risk.

## Introduction

Falls represent a major public health concern, imposing a dual burden of physical injury and psychological distress that can severely compromise functional independence and quality of life (1, 2). This challenge is particularly acute amid an aging global population, where the proportion of older adults continues to rise, and the number of individuals over 65 is projected to more than double by 2050 (3, 4). As this population grows, fall-related injuries threaten both individual wellbeing and healthcare systems worldwide (5–7).

The problem of falls extends beyond aging demographics. Sedentary lifestyles prevalent in modern societies contribute to sarcopenia, reduced musculoskeletal strength, and impaired coordination, elevating fall risk among both older and middle-aged adults (8, 9). Epidemiological evidence demonstrates increasing prevalence of balance disorders even among healthy middle-aged populations (8), highlighting an emerging public health concern that will intensify as this cohort ages (6). Specifically, occupational falls represent an additional and often underappreciated dimension of this public health challenge, constituting one of the leading causes of work-related injury and disability among working-age adults (10–12). Falls in occupational settings account for a substantial proportion of workplace injuries and long-term disability globally, with workers in industries such as construction, manufacturing, healthcare, education, and agriculture particularly exposed to elevated fall risk (12). Beyond the direct physical consequences, occupational falls impose considerable economic burden through lost productivity, prolonged absence from work, and healthcare utilisation (11), highlighting that fall risk and its consequences are not confined to older adults but are relevant across the adult lifespan.

Compounding these broader risk factors, modern architectural and cultural norms have reduced floor interaction through the elimination of floor-related activities, now replaced by prolonged chair sitting (13, 14). This shift eliminates activities of daily living such as cross-legged sitting, kneeling, and squatting, movements that were once performed regularly on the floor that supported the musculoskeletal and cardiovascular fitness necessary for healthy aging and fall prevention (15). Evidence from population groups who commonly assume floor-based postures during rest demonstrates significantly higher lower-limb muscle activity, particularly in the soleus, vastus lateralis, and tibialis anterior, compared to chair sitting (16). These findings suggest that habitual lower-limb muscle engagement may be reduced when floor-based postures are replaced by prolonged chair sitting. However, whether such differences translate into meaningful changes in strength, metabolic health, balance capacity, or fall resilience remains to be established through longitudinal research.

Beyond these physiological effects, this lack of floor interaction in urbanized societies may foster uncertainty and hypervigilance toward the floor (17). When individuals do not regularly sit on the floor, they lose the strength, flexibility, and coordination required to rise independently (18), thereby creating both physical and psychological barriers. Research demonstrates that individuals unable to rise independently from the floor experience poorer health outcomes including higher likelihood of morbidity and all-cause mortality (19, 20), as well as elevated concern of falling (21). To address this multifaceted issue, novel therapeutic interventions are needed that target both physiological and psychological barriers associated with falls in a sustainable manner, supporting individuals as they age to independently maintain function throughout later life while promoting healthy aging.

Existing fall management interventions typically target falls symptomatically (22–24), offering physical training (balance and strength exercises) and psychological support (cognitive-behavioral strategies). While these evidence-based approaches demonstrate some benefit, their effects on psychological outcomes associated with falls remain modest (25, 26). More fundamentally, current programs focus primarily on fall avoidance and seldom consider individuals’ relationship with the floor itself or provide structured training in safe post-fall management. This represents a significant gap in care, as avoiding falls entirely is impractical given that age-related physiological decline will eventually lead to unintentional falls. A pressing need therefore exists for rehabilitation programs that help individuals become more confident and comfortable with the floor environment. This can be achieved through activities that involve moving on and interacting with the floor and through role-playing activities simulating fall scenarios, particularly in high fall-risk settings. Such interventions may reduce uncertainty and encourage individuals to incorporate floor-related movements into daily life, leading to long-term, sustainable improvements in mobility and independence. Moreover, developing contingencies for independent fall management is equally important, ensuring that if individuals do fall in the future, they possess both the capability and knowledge to recover independently. This component is especially important given that research demonstrates healthy individuals rarely receive instruction in floor-to-standing strategies from healthcare professionals, with physiotherapists and nurses typically reserving such training for patients only after a fall has occurred i.e., when they require emergency care and hospitalization (27).

To address these challenges, the Floor-Hugging Intervention (Floor-HI) was developed around two key components: (1) controlled exposure to fall-risk environments and fall positions, and (2) systematic training in post-fall recovery skills, including both supported and independent floor-to-standing transitions (17). This dual approach aims to simultaneously strengthen physical capabilities and build psychological resilience. The theoretical foundation underlying Floor-HI is grounded in the Uncertainty and Anticipation Model (28), positing that by improving floor exposure and developing post-fall contingencies, the intervention can reduce uncertainty about future fall threats. This reduction in uncertainty could alleviate maladaptive responses while improving fall self-efficacy and preparedness.

This exploratory study aimed to primarily evaluate the feasibility, acceptability, and safety of Floor-HI in community-dwelling middle-aged adults. Secondary objectives included exploratory examination of changes in concern of falling, postural control, floor-rise capacity, turning ability, and health-related quality of life. These findings will inform whether the protocol requires modification and whether progression to a larger controlled trial is warranted.

## Methods

### Study design

**Figure 1:**
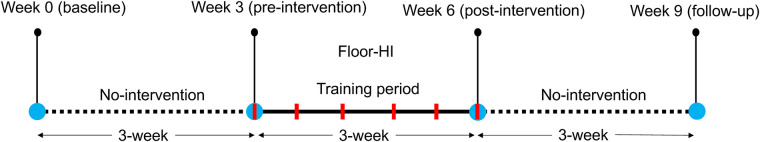
Timeline of the sequential pre-post study design for the floor-hugging intervention (Floor-Hi). Blue circles indicate assessment time points, while red lines represent individual Floor-HI training sessions conducted between weeks 3 and 6.

The exploratory study employed a multiple pre- and post-test design (Figure 1), where participants were assessed at four time points: baseline (week 0), pre-intervention (week 3), post-intervention (week 6), and follow-up (week 9). The study was part of a pre-registered trial registered in the ClinicalTrials.gov database (NCT06815809).

### Recruitment

Participants were recruited consecutively, with data initially collected between February 2025 and August 2025. Following peer review, data collection was extended through March 2026 to increase sample size and statistical power. All results reported in this manuscript reflect the complete dataset. Participants were included if they were between 18 and 64 years of age, could understand English or Swedish, were free from any musculoskeletal or neurological disorders, and were not suffering from any psychiatric conditions or moderate to severe cognitive impairments. Participants were also excluded if they were engaging in any additional fall-prevention interventions during their study participation. The study received ethical approval from the Swedish Ethical Review Authority (Dnr: 2024-07271-01). Written informed consent was obtained from all participants, and the study was conducted at the University premises.

### Floor-HI description

The Floor-HI intervention was developed by Ghai and Ghai (17), and was delivered on an individual basis by the author, a trained physiotherapist with doctoral-level training and academic experience in rehabilitation research. Training sessions occurred two times per week for three consecutive weeks, resulting in a total of six one-hour sessions. The intervention was performed on sports mats (NordicFighter AB, Sweden) and employed a progressive surface-reduction protocol that systematically decreased mat thickness to simulate realistic fall environments and enhance participants’ confidence and self-efficacy in fall recovery. Mat thickness was reduced progressively across the three weeks, from 40 mm in week one to 20 mm in week two, concluding on an unpadded hard floor in week three.

**Figure 2:**
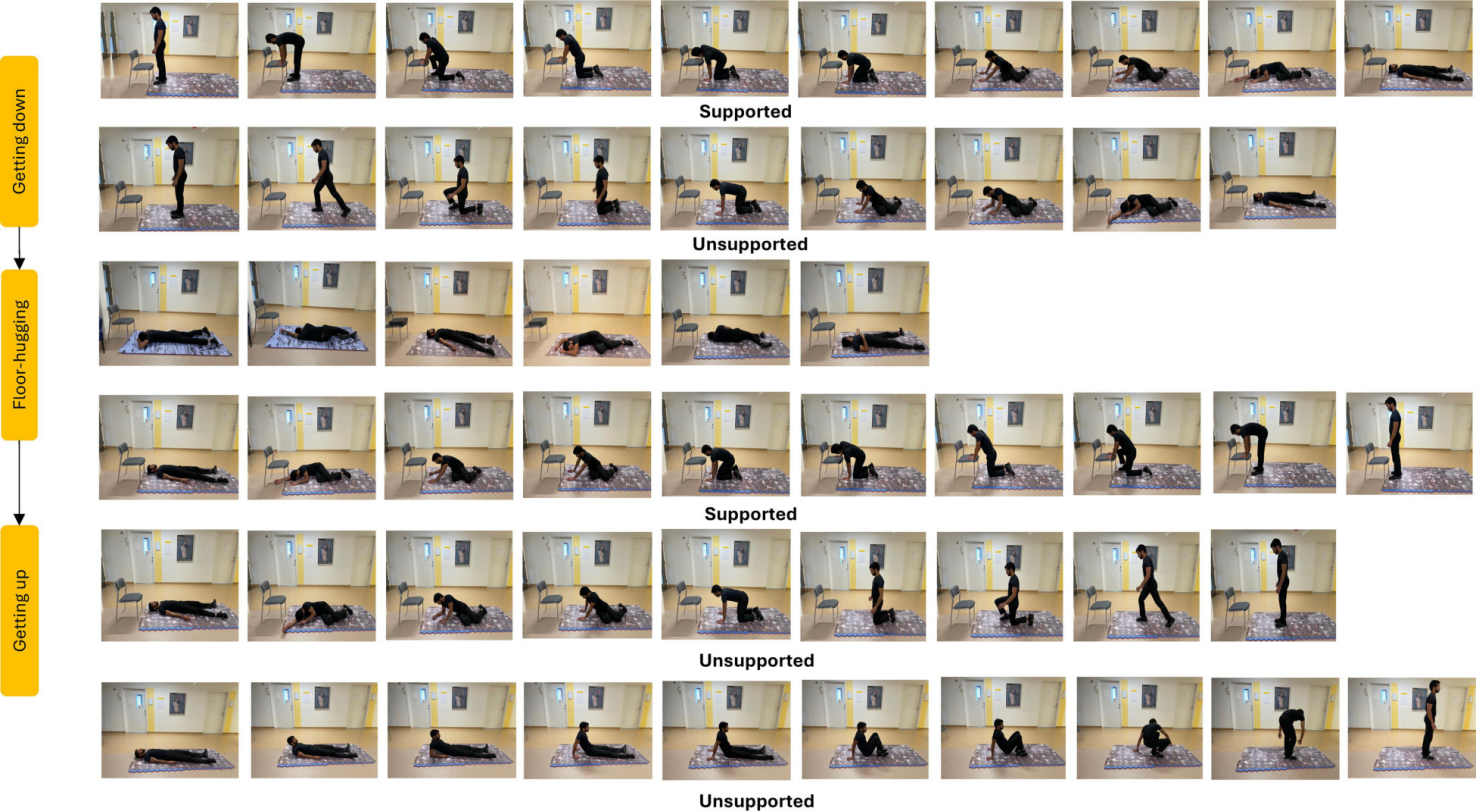
Steps of the floor-hugging intervention include transitioning to the floor, performing floor-hugging movements, and practicing floor-rise techniques using both supported (with furniture) and unsupported methods.

**Figure 3:**
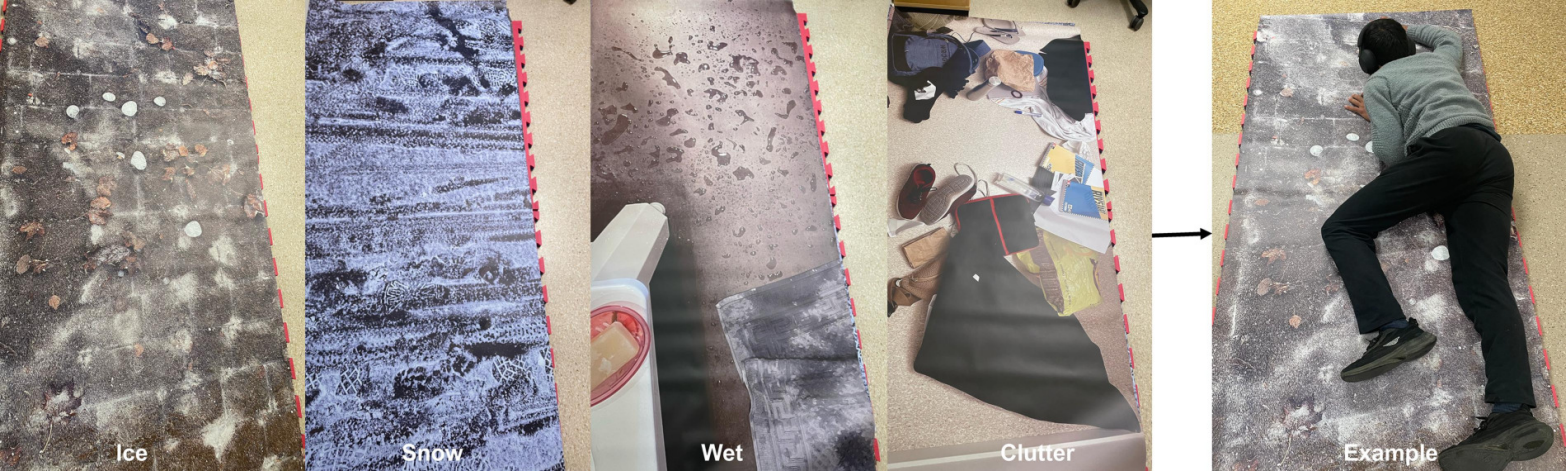
High fall-risk printed surfaces (ice, snow, wet, clutter) used in training environments to simulate ecologically salient outdoor and indoor hazards.

Each training session comprised three phases (Figure 2) designed to comprehensively address fall recovery skills. Each one-hour session was structured to allocate approximately 15 min to the floor-descent phase, 20 min to floor-position exposure and functional exercises, and 25 min to floor-rising practice, with timing adjusted based on individual participant needs and progression. The initial phase focused on teaching participants to lower themselves to the floor using a backward-chaining approach, initially incorporating external support from a chair before progressing to an adapted unsupported version as participants gained comfort and confidence. A sturdy, non-wheeled chair secured against a wall or fitted with a non-slip mat was used to ensure safety during supported descents. The second phase required participants to assume various positions including supine, prone, side-lying, and semi-prone while lying on printed floors imitating environments such as ice, snow, clutter, or wet surfaces (Figure 3). These surfaces were employed to provide action-relevant environmental context, allowing individuals to safely experience high-risk fall environments they might typically avoid, thereby potentially facilitating real-world transferability.

**Figure 4:**
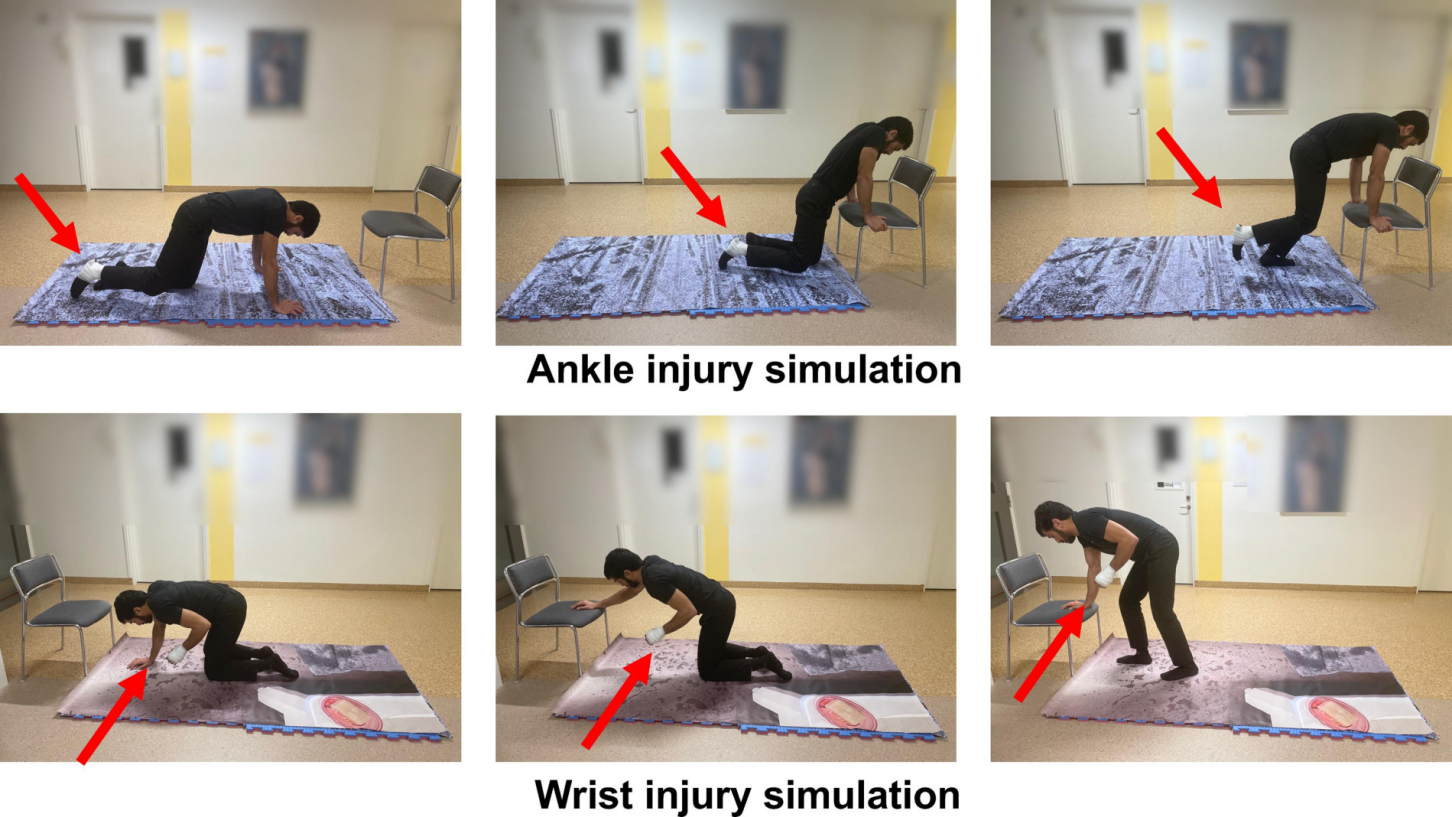
Simulated ankle and wrist injuries during the adapted floor-rise technique (the red arrows indicate the injury simulation area, which was bandaged to remind participants of the simulated injury).

During this phase, participants practiced observing their surroundings before being invited to imagine that they had fallen on that surface while employing deep-breathing relaxation techniques to alleviate fall-related anxiety. The third phase focused on floor rising and initially utilized a forward-chaining strategy with assistive furniture support, with participants advancing to an unsupported rise method as their balance and confidence improved. This unsupported method comprised an adaptation of the forward chain approach where participants transitioned directly from the all-fours position through kneeling to lunging and finally to standing. Participants with sufficient balance and strength capabilities progressed to the seven-component method described by Schwickert and colleagues (29), encompassing lying, initiating, positioning, supporting, elevation, stabilization, and standing phases. During this phase, role-playing adaptations were incorporated in which participants imagined having sustained injuries to specific body parts, such as an ankle, wrist, or lower back (Figure 4), and practiced adapting their floor-rise techniques to accommodate these simulated injuries.

**Figure 5:**
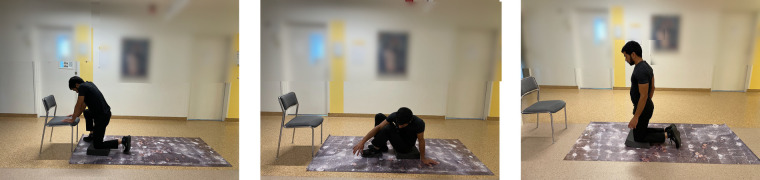
Use of foam wedge to improve comfort and adaptation during floor transitions.

Throughout the intervention, targeted adaptations (Figure 5) were integrated between and within training sessions to personalize the program. Foam wedges were placed under the knee during floor kneeling to assist participants with unsupported floor rises. The progressive surface reduction protocol described above further supported this individualisation, habituating participants to real-life fall conditions including floor stiffness against bony prominences.

**Figure 6:**
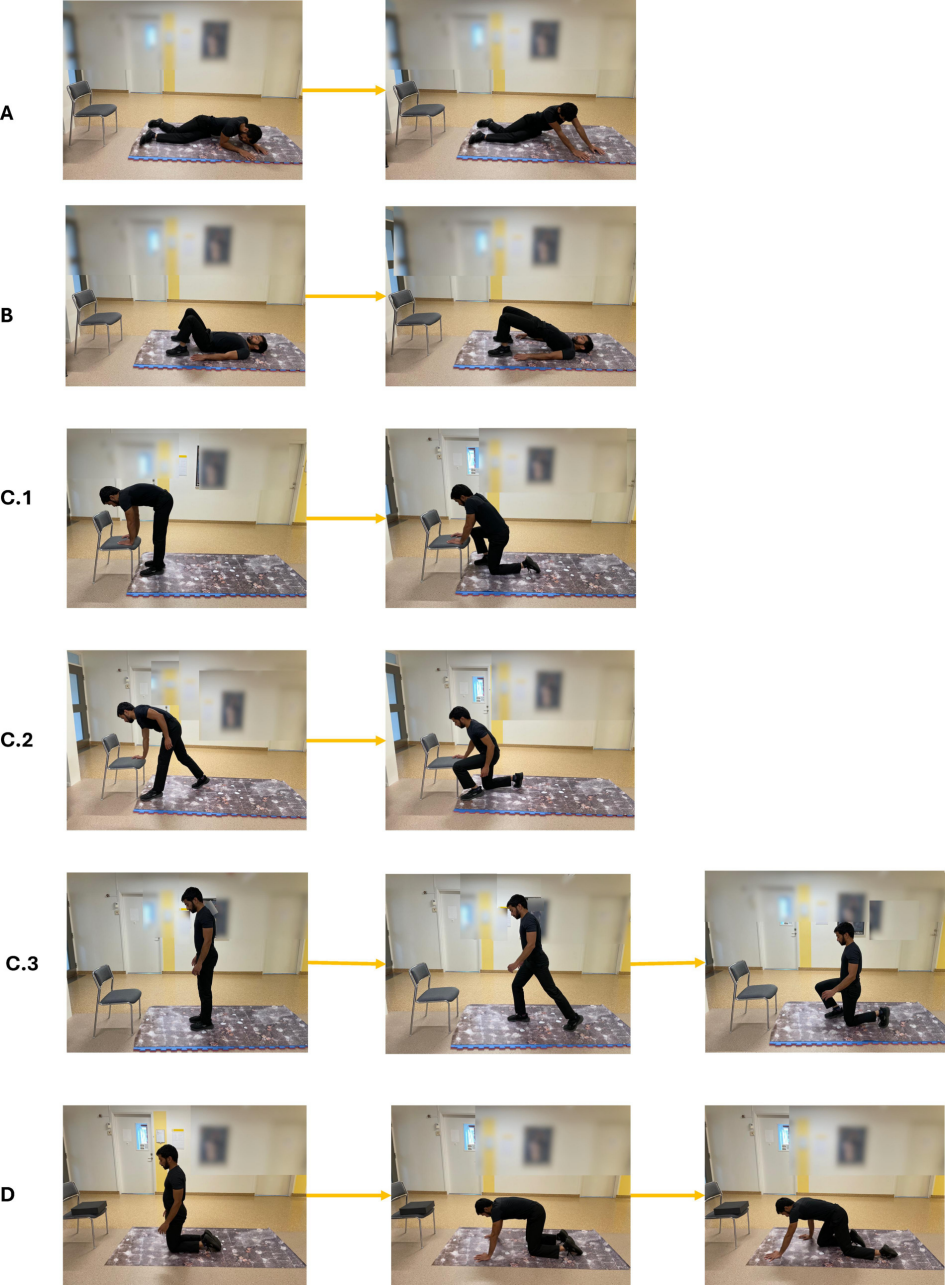
Exercises and activities conducted as part of the floor-HI program: **(A)** maintaining the mermaid position for upper extremity strengthening, **(B)** bridging, (C.1) Supported lunge with double hand support, (C.2) Semi-supported lunge with one hand support, (C.3) Unsupported lunge with no hand support, and **(D)** Maintaining positions on hard surface: kneeling, all-fours position, and all four locomotion.

Participants also performed a series of functional exercises during each session (Figure 6), comprising supported and unsupported lunges (10 repetitions per side, twice per session) to develop lower-extremity strength essential for controlled descent and ascent movements. An all-fours position (60 s, twice per session) was held for progressively longer durations to accustom the knees and hands to sustained loading on the patellar and palmar surfaces. Prolonged kneeling (60 s, twice per session), particularly during week three, habituated the knee joints to direct contact with hard surfaces. A unilateral arm-support position (“mermaid” position, 60 s per side, twice per session) was used to enhance upper-extremity strength and endurance. These functional exercises were interspersed throughout the session, building the physical capacity necessary for safe floor transitions while also providing active recovery between floor-position exposures. Where difficulty was encountered during the kneeling-to-standing transition, low-density foam pads were placed beneath the knee during the elevation phase, providing compressible support without diminishing the functional challenge.

### Feasibility outcomes

Feasibility, acceptability, and safety of Floor-HI were evaluated through the following:

1. Recruitment: The recruitment rate was assessed as the proportion of individuals screened who met eligibility criteria and subsequently enrolled, reflecting the accessibility and appeal of the intervention to the target population.
2. Retention: The retention rate was assessed as the proportion of enrolled participants who completed the full study protocol, reflecting the intervention's ability to sustain engagement across the study period.
3. Adherence: The adherence rate was assessed as the proportion of enrolled participants who attended all of the scheduled training sessions and follow-up assessments, reflecting the degree to which participants were able to engage with the intervention as intended.
4. Intervention Acceptability: Acceptability was evaluated using the Theoretical Framework of Acceptability (TFA) questionnaire, covering seven constructs including intervention coherence, burden, and self-efficacy (30).
5. Intervention-related safety: The safety of Floor-HI was monitored through two approaches. Adverse events were documented throughout the intervention phase (Weeks 3–6) using the National Institute on Aging Adverse Event Form. Additionally, falls were recorded daily using a fall diary capturing each incident's cause, location, and consequences (31), which were reviewed at each training session and collected at the Week 9 follow-up.

### Clinical outcomes

Outcomes were assessed at four time points (Weeks 0, 3, 6, and 9) and are summarised in Table 1. Minimal Important Change (MIC) values have been reported where available, and available threshold values including Minimal Clinically Important Difference (MCID) or Minimal Detectable Change (MDC) have been reported where MIC values were unavailable. As these values were not found in the existing literature for healthy middle-aged adults, reported values from patient populations were considered for reference.

1. Perceived concern of falling: Assessed using the Falls Efficacy Scale-International (FES-I) (32), a 16-item questionnaire measuring concern about falling during daily activities. Total scores ranged from 16 to 64, with higher scores indicating greater concern of falling. No MIC has been established for the FES-I (33), a MCID of 5.5 points was therefore considered (34).
2. Ability to Rise from the Floor: The Sitting Rising Test (SRT) assessed the ability to transition between floor sitting and standing with minimal support (35). Scores ranged from 0 to 10, with deductions made for reliance on support or unsteady movements, and higher scores reflecting better performance. Each one-point increase has been associated with a 21% improvement in survivability, suggesting clinical relevance, though no MIC has been established (35).
3. Balance and Postural Control: The Mini Balance Evaluation Systems Test (Mini-BESTest) assessed static and dynamic postural stability through 14 balance-related tasks (36). Total scores ranged to 28, with higher scores indicating superior balance. An MIC of 4 points was considered (37).
4. Turning Performance: The 360° Turn Test measured the time and number of steps required to complete a full circle turn on the dominant and non-dominant side (38), serving as a practical indicator of coordination, dynamic balance, and turning agility. No MIC has been established, though MDC values of 1.49 s (dominant) and 1.53 s (non-dominant) have been reported (39).
5. Health-Related Quality of Life (HRQoL): The RAND 36-Item Health Survey assessed HRQoL across eight domains including physical functioning, role limitations, bodily pain, emotional well-being, social functioning, energy, and general health perceptions (40). Domain scores ranged from 0 to 100, with higher scores representing better perceived health. An MCID of 3–5 points was considered (40), as no MIC has been established.

**Table 1:** Assessment schedule for outcome measures at each time point.

Measures	Week 0	Week 3	Week 6	Week 9
Feasibility outcomes				
Recruitment	**✓**			
Retention				**✓**
Adherence			**✓**	
Fall diary	**✓**	**✓**	**✓**	**✓**
TFA			**✓**	
Adverse event log			**✓**	
Clinical outcomes				
FES-I	**✓**	**✓**	**✓**	**✓**
Mini-BESTest	**✓**	**✓**	**✓**	**✓**
SRT	**✓**	**✓**	**✓**	**✓**
360° turn test	**✓**	**✓**	**✓**	**✓**
RAND-36	**✓**	**✓**	**✓**	**✓**

FES-I, falls efficacy scale-international; Mini-BESTest, mini balance evaluation system test; SRT, sitting rising test; RAND-36, RAND 36-item health survey; TFA, theoretical framework of acceptability.

### Data analysis

Participant characteristics and outcome measures were summarised using descriptive statistics and reported as median and range, reflecting the small sample size and non-normal data distribution. Changes across four time points (Weeks 0, 3, 6, and 9) were examined using the Friedman test for repeated measures. Where a significant overall effect was detected, *post-hoc* pairwise comparisons were performed using Wilcoxon signed-rank tests with Bonferroni correction applied across three consecutive intervals (Weeks 0–3, 3–6, and 6–9), yielding an adjusted significance threshold of *p* < 0.017. Effect sizes were calculated for all Wilcoxon tests and interpreted as small (0.1), medium (0.3), or large (0.5). All analyses were conducted in IBM SPSS Statistics (Version 29).

## Results

### Feasibility outcomes

**Figure 7:**
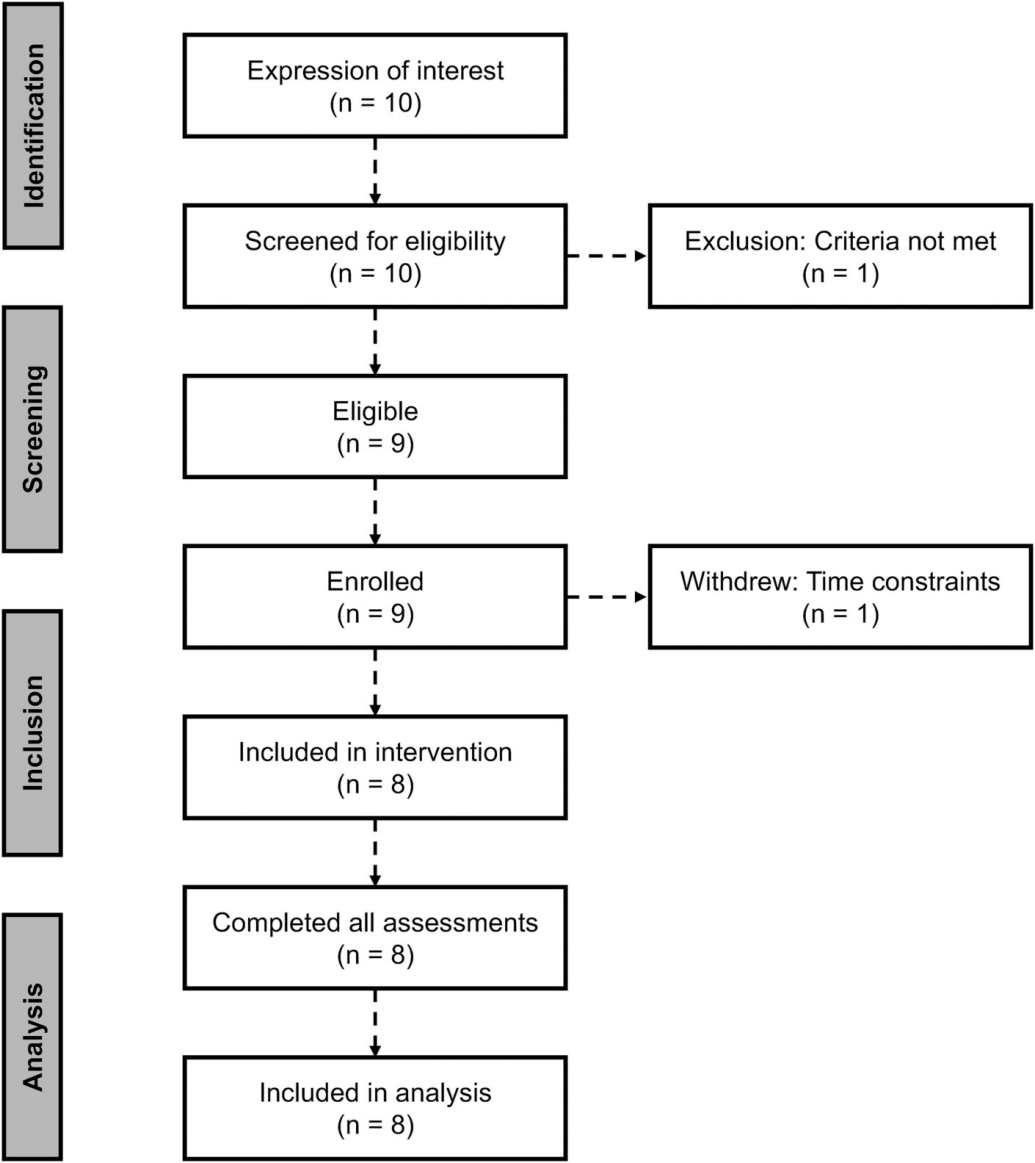
Participant inclusion flow chart.

The feasibility of the Floor-HI intervention was evaluated in terms of recruitment, retention, and adherence to the intervention protocol. A total of ten individuals expressed interest and were screened for eligibility. One was excluded for not meeting the inclusion criteria (>64 years), and the remaining nine were successfully enrolled, yielding a recruitment rate of 100%. One further participant withdrew after completing the baseline assessment due to time constraints and was therefore excluded from further analyses. The remaining eight participants completed the study, resulting in a retention rate of 89%. Adherence to the intervention protocol was 100%, with all eight participants attending every scheduled training session and completing assessments at all four time points (Week 0, Week 3, Week 6, and Week 9) (Figure 7).

The intervention was delivered as planned, with no deviations from the protocol other than occasional rescheduling of individual training sessions. No interruptions to the overall training schedule were recorded. Participants were able to complete all components of the intervention, including floor-based exercises and progressive exposure to reduced surface support, indicating that the protocol was both practical and implementable in this population.

### Demographics

Eight healthy, community-dwelling, middle-aged adults (3 males, 5 females; aged 31–55 years) participated in the Floor-HI study. Demographic characteristics are presented in Table 2.

**Table 2:** Demographic characteristics of included participants.

Characteristics	Median (range), or *N*%
Total participants	8
Sex	
Female	5
Male	3
Age (years)	44 (31–55)
Physical activity per week (hours)	
0–1 h	1
2–5 h	6
6–10 h	1
Floor-time daily (minutes)	
<15 min	7
15–30 min	1
Fall history (past 1 year)	
Yes	3
No	5

### Clinical outcomes

Summary statistics (median and range) for all clinical outcomes are presented in Table 3. Additional domain-specific data for the Mini-BESTest and the Sitting Rising Test are provided in Supplementary Tables S1, S2.

**Table 3:** Clinical outcome measures at each time point (median, range).

Measures	Week 0	Week 3	Week 6	Week 9
Fall efficacy scale-international	22.5 (18.0–33.0)	19.0 (17.0–24.0)	19.0 (17.0–25.0)	17.5 (16.0–25.0)
Mini-BESTest	20.5 (19.0–24.0)	22.0 (18.0–23.0)	27.0 (25.0–28.0)	26.5 (25.0–28.0)
Sitting rising test	6.5 (3.5–9.0)	7.0 (5.0–9.0)	9.3 (6.5–10.0)	8.8 (7.5–10.0)
360° turn test				
Duration (seconds)				
Dominant	2.8 (2.0–3.7)	2.9 (1.7–4.4)	2.1 (1.5–2.5)	2.3 (1.5–2.3)
Non-dominant	3.0 (1.9–4.4)	2.8 (1.8–3.9)	2.1 (1.4–2.9)	2.1 (1.5–3.0)
Steps (*N*)				
Dominant	7.0 (5.0–10.0)	7.0 (5.0–8.0)	5.0 (4.0–6.0)	4.5 (4.0–6.0)
Non-dominant	7.0 (5.0–10.0)	6.5 (4.0–9.0)	4.5 (3.0–6.0)	5.0 (3.0–5.0)
RAND-36				
Physical function	95.0 (50.0–100.0)	95.0 (90.0–100.0)	95.0 (75.0–100.0)	95.0 (80.0–100.0)
Role-physical	100.0 (100.0–100.0)	100.0 (50.0–100.0)	100.0 (75.0–100.0)	100.0 (75.0–100.0)
Role-emotional	100.0 (33.3–100.0)	100.0 (66.6–100.0)	100.0 (0.0–100.0)	100.0 (0.0–100.0)
Vitality	60.0 (45.0–85.0)	75.0 (50.0–85.0)	77.5 (35.0–85.0)	72.5 (35.0–100.0)
Mental health	76.0 (64.0–100.0)	78.0 (64.0–96.0)	80.0 (60.0–100.0)	76.0 (56.0–100.0)
Social functioning	100.0 (75.0–100.0)	100.0 (75.0–100.0)	100.0 (62.5–100.0)	100.0 (62.5–100.0)
Bodily pain	90.0 (77.5–100.0)	90.0 (77.5–100.0)	90.0 (80.0–90.0)	90.0 (67.5–100.0)
General health	72.5 (70.0–75.0)	75.0 (55.0–85.0)	75.0 (60.0–85.0)	75.0 (60.0–85.0)
Theoretical framework of acceptability				
Affective attitude	–	–	5.0 (4.0–5.0)	–
Burden	–	–	4.0 (2.0–4.0)	–
Ethicality	–	–	5.0 (3.0–5.0)	–
Perceived effectiveness	–	–	4.0 (3.0–5.0)	–
Intervention coherence	–	–	4.5 (4.0–5.0)	–
Self-efficacy	–	–	4.0 (4.0–5)	–
Opportunity costs	–	–	4.0 (2.0–5.0)	–
General acceptability	–	–	5.0 (4.0–5.0)	–

### Fall efficacy scale-international

During the pre-intervention phase, FES-I scores decreased from Week 0 (median, range: 22.5, 18–33) to Week 3 (19.0, 17–24), though this reduction did not reach statistical significance across the four time points [*χ*²(3) = 5.40, *p* = .145]. Notably, Week 0 assessments were conducted during winter conditions (January-February), whereas Week 3 assessments took place in spring (March), which may have contributed to the observed trend, although this was not formally assessed.

### Mini-BESTest

A significant effect of time was observed [*χ*²(3) = 20.68, *p* < .001]. Pairwise comparisons showed no change during the pre-intervention phase (Weeks 0–3, *p* = .39). In contrast, postural stability improved during the intervention phase (Weeks 3–6, *p* = .011, r = 0.90), exceeding the MIC threshold. These improvements were maintained at follow-up (Weeks 6–9, *p* = .257).

### Sitting rising test

A significant effect of time was also observed for SRT scores [*χ*²(3) = 22.42, *p* < .001]. Floor-rise ability did not change during the pre-intervention phase (Weeks 0–3, *p* = .042, exceeding the Bonferroni-corrected threshold of *p* < .017). However, floor-rise ability improved during the intervention phase (Weeks 3–6, *p* = .011, r = 0.90), exceeding the minimal important change threshold. These gains were sustained at follow-up (Weeks 6–9, *p* = .564).

### 360° turn test

#### Duration

Turning duration changed significantly over time for both dominant [*χ*²(3) = 19.99, *p* < .001] and non-dominant sides [*χ*²(3) = 16.18, *p* = .001]. No changes were observed during the pre-intervention phase (Weeks 0–3; dominant: *p* = .674; non-dominant: *p* = .611). During the intervention phase (Weeks 3–6), duration decreased on both the dominant (*p* = .012, r = 0.89) and non-dominant sides (*p* = .017, r = 0.84), with improvements exceeding MDC values. Performance remained stable at follow-up (Weeks 6–9; dominant: *p* = .496; non-dominant: *p* = .276).

#### Steps

A similar pattern was observed for step count, with changes over time for both dominant [*χ*²(3) = 19.77, *p* < .001] and non-dominant sides [*χ*²(3) = 15.78, *p* = .001]. Step count remained unchanged during the pre-intervention phase (Weeks 0–3; dominant: *p* = .666; non-dominant: *p* = .288). During the intervention phase (Weeks 3–6), step count decreased on the dominant side (*p* = .010, r = 0.91), indicating improved turning efficiency, while the reduction on the non-dominant side did not reach statistical significance after correction (*p* = .028, r = 0.78). No changes were observed at follow-up (Weeks 6–9; dominant: *p* = .564; non-dominant: *p* = 1.000).

#### RAND-36

No changes over time were observed across any RAND-36 domains, including physical function [*χ*²(3) = 2.82, *p* = .421], role-physical [*χ*²(3) = 1.29, *p* = .733], role-emotional [*χ*²(3) = 2.20, *p* = .532], vitality [*χ*²(3) = 3.51, *p* = .320], mental health [*χ*²(3) = 0.08, *p* = .994], social functioning [*χ*²(3) = 2.40, *p* = .494], bodily pain [*χ*²(3) = 0.40, *p* = .941], and general health [*χ*²(3) = 1.00, *p* = .801].

### Theoretical framework of acceptability

Overall acceptability of the intervention was high (median, range: 5, 4.0–5.0), with participants providing favourable ratings across all TFA domains (Table 3), including affective attitude, ethicality, and perceived effectiveness, indicating that the intervention was viewed positively and perceived as manageable throughout the study period.

### Adverse events

#### Fall diary

One fall was reported during the pre-intervention phase (Weeks 0–3), in which a single participant slipped on an icy pavement. No falls occurred during the intervention (Weeks 3–6) or follow-up (Weeks 6–9) phases.

#### Intervention-related adverse events

Adverse events were monitored during the intervention phase (Weeks 3–6, Table 4). All eight participants reported at least one adverse event. The most common event was muscle soreness (*n* = 8). Two participants reported pain (*n* = 2), and one participant reported knee bruising (ecchymosis) sustained during floor transfer exercises performed on a hard surface with no associated skin breakdown. All adverse events were mild, transient, and resolved without medical intervention. No serious adverse events were recorded.

**Table 4:** Details of adverse events.

Adverse events	Week 0–3	Week 3–6	Week 6–9
Fall diary			
Number of falls	1	0	0
Number of individual	1	0	0
Intervention-related adverse events (*N*)			
Soreness	–	8	–
Pain	–	2	–
Bruising	–	1	–

## Discussion

This exploratory study provides evidence supporting the feasibility of Floor-HI as a novel multicomponent intervention targeting both physical and psychological aspects of fall risk. The findings demonstrate that the intervention can be successfully implemented in community-dwelling middle-aged adults, with high feasibility and acceptability, minimal adverse events, and potential improvements in key functional domains relevant to fall prevention.

### Feasibility & acceptability

The results indicate that Floor-HI is feasible to deliver in community-dwelling middle-aged adults. Of the nine participants enrolled, eight completed the study, yielding a retention rate of 89%, while adherence to the intervention protocol was 100%. All participants attended scheduled sessions and completed outcome assessments, suggesting that the intervention was practical and manageable within the study context.

Acceptability was consistently high across all domains of the TFA, with median scores across domains ranging from 4.0 to 5.0 out of 5 and overall acceptability rated at 5/5. These findings indicate that participants perceived the intervention as appropriate, manageable, and beneficial. Notably, high acceptability was maintained despite the physical demands of floor-based training, as reflected in the burden subdomain. This suggests that the progressive design and individualized adaptations were effective in supporting engagement and tolerance.

Beyond quantitative ratings, a compelling indicator of the intervention's value emerged from the intervention coherence domain of the TFA questionnaire. Notably, five of the eight participants spontaneously requested additional training materials (i.e., intervention protocols) at the end of the study, expressing a desire to continue the intervention independently and maintain their newly acquired skills. This unsolicited request for continuation materials represents a strong endorsement of the intervention's perceived value, suggesting that participants experienced meaningful benefits they wished to sustain beyond the formal study period. Furthermore, this finding supports the potential for long-term adherence and sustained benefits of Floor-HI in real-world settings, although this will require confirmation through studies with extended follow-up periods.

### Safety profile

The safety profile of Floor-HI was characterised by a low number of adverse events, all of which were mild and transient in nature. A total of three categories of adverse events were recorded across all eight participants: muscle soreness (*n* = 8), pain during hard-surface contact tasks such as kneeling or weight-bearing through the hands (*n* = 2), and localised ecchymosis over the tibial tuberosity sustained during floor transfer practice (*n* = 1). All events were mild and transient in nature, resolving without medical intervention, and no serious adverse events were recorded. The most common event, muscle soreness and discomfort associated with floor contact and transitional movements, was anticipated given the nature of the training and the relatively low baseline physical activity levels of participants, and is consistent with expected training-related adaptations such as delayed-onset muscle soreness.

Pain during tasks involving direct contact with hard surfaces, such as kneeling or weight-bearing through the hands, was reported by two participants. These issues were effectively managed through simple modifications, including the use of supportive equipment (e.g., foam), enabling participants to continue the intervention without interruption. One participant reported localized ecchymosis over the tibial tuberosity sustained during floor transfer practice on a hard surface, with no associated skin breakdown. Importantly, no serious adverse events were observed across the cohort. Collectively, these findings support the safety and tolerability of Floor-HI in this population.

## Functional outcomes

### Concern of falling

FES-I scores remained stable across all assessment periods throughout the intervention (i.e., between Weeks 0 and 9). Several factors may explain this lack of change. First, the minimal change in concern of falling may be attributed to the relatively low pre-intervention concern among all eight participants, which may have created a floor effect limiting the potential for meaningful improvement. A previous study by Cox and Williams (41) also reported a lack of impact of floor rise training and fall education on concern of falling outcomes.

Second, when asked to elaborate on their views regarding the intervention as part of the TFA questionnaire, the majority of participants (i.e., 5/8 participants) indicated that Floor-HI made them more aware of fall risk. For instance, they reported that prior to participating in Floor-HI, they had not consciously thought about falls and had overestimated their ability to rise from the floor. However, after completing the assessments and intervention, they became more aware of their true physical capabilities, which may have slightly increased their concern about falling.

Importantly, the FES-I measures concern about falling during activities, whereas the TFA, particularly its self-efficacy construct, captures confidence in one's ability to perform intervention-related behaviors. These represent related but distinct psychological constructs. From the perspective of Protection Motivation Theory, concern about falling (threat appraisal) and confidence in managing floor transitions (coping appraisal) represent distinct but complementary processes (42). Here, excessively low concern about falling may reflect risk underestimation, whereas improved confidence in one's ability to respond effectively to a fall may enhance preparedness and resilience. Therefore, the observation of relatively stable FES-I scores alongside high self-efficacy ratings on the TFA can be interpreted as participants maintaining appropriate level of concern about fall risk while simultaneously improving their confidence in managing floor transitions and post-fall recovery. In this context, stable concern coupled with improved self-efficacy reflects adaptive changes across complementary psychological domains, both of which could be meaningful outcomes in fall management.

Third, the framing of FES-I items may have also influenced outcomes. The FES-I assesses concern about falling with items determining the level of concern about falling during specific activities (i.e., “How concerned are you about falling when..”). Due to participants’ increased awareness of fall-related injuries, high-risk environments, and their own functional limitations, expressed concern might increase even as functional capacity improves. However, using instruments framed differently, such as the Activities-specific Balance Confidence (ABC) Scale, which assesses confidence rather than concern (i.e., “How confident are you in carrying out these activities?”), may have yielded different outcomes. Such measures may better capture improvements in fall-related self-efficacy observed in this study. Ting, Ho (43) similarly noted that although FES-I and ABC demonstrate high congruency, they evaluate different psychological constructs of falls. This highlights an important methodological consideration when selecting outcome measures to capture multiple dimensions of concern of falling.

### Balance performance

The most consistent and clinically meaningful change was observed in static and dynamic balance performance outcomes assessed by the Mini-BESTest. Initially, scores remained stable during the pre-intervention phase (Weeks 0–3, *p* = .39), followed by a significant improvement during the intervention phase (Weeks 3–6, *p* = .011, r = 0.90), exceeding the MIC threshold (44). These gains were maintained at follow-up (Weeks 6–9), with no significant decline observed (*p* = .26).

Specifically, the largest improvements within the Mini-BESTest domains were observed in reactive postural control (Week 3–6 improvement: 2 points) and dynamic gait (3 points) domains (Supplementary Table S2). The observed improvements in reactive postural control are clinically meaningful, as this domain reflects the body's ability to recover from unexpected perturbations and maintain stability during challenging conditions. This enhancement suggests that the intervention may have targeted underlying neuromuscular mechanisms responsible for rapid postural adjustments, which are crucial for preventing accidental falls during activities of daily living. Simultaneously, improvements in dynamic gait performance further support the intervention's potential in addressing complex, multi-component balance tasks that closely reflect real-world mobility demands. The maintenance of these gains at the three-week follow-up period is particularly encouraging from a clinical perspective, as it indicates that the intervention resulted in adaptations rather than transient performance effects.

Similarly, improvements in 360° turning performance also represent clinically meaningful findings, with participants demonstrating significant reductions between Weeks 3 and 6 in turn duration (dominant: *p* = .012, non-dominant: *p* = .017) and step count (dominant: *p* = .010) during the turning task. This dual improvement indicates enhanced neuromuscular coordination and improved dynamic stability during rotational movements. The reduction in step count suggests that participants developed more efficient movement strategies, requiring fewer corrective steps to maintain balance during turning. At the same time, the decreased completion time indicates improved confidence and fluidity in executing the turn, reflecting enhanced motor control and reduced movement hesitation. These performance gains are particularly relevant from a fall prevention perspective, given the inherently challenging nature of turning tasks. Turning activities have been consistently identified as high-risk movements associated with falls and hip fractures in community-dwelling adults (45, 46). This elevated risk is largely attributable to the biomechanical demands of turning, which inherently create instability due to the asymmetric nature of the movement, where one leg takes shorter steps while the other takes longer steps to navigate the rotational trajectory (47, 48). This asymmetric stepping pattern challenges the body's ability to maintain postural control, particularly when combined with momentum generation and the need for coordinated multi-segmental control.

### Floor-rise ability

Floor-rise ability, as assessed by the SRT, showed significant improvement across participants (Weeks 3–6: *p* = .011), with scores improving by a median difference of 2.3 points post-intervention. This finding is particularly relevant given that the inability to rise from the floor independently is a strong predictor of functional decline, institutionalization, and all-cause mortality (19, 49).

Moreover, an important clinical observation was that all eight participants demonstrated instability during baseline performance, particularly during the sitting-down phase, where participants exhibited an uncontrolled descent into a seated position rather than a controlled lowering. This issue resolved in all participants by the end of the study. The resolution of movement instability represents a crucial functional improvement, as unstable movement patterns during floor-to-standing transitions indicate compromised neuromuscular control that can substantially increase fall risk.

Importantly, the developers of the SRT have demonstrated that each one-point increase in score is associated with a 21% improvement in survival (15). While this association should not be interpreted causally, it underscores the potential clinical relevance of improvements in floor-rise ability. The gains observed in SRT performance in this study may therefore reflect broader enhancements in integrated physical function, including improved lower extremity strength, trunk stability, flexibility, and neuromuscular coordination, key components of healthy aging.

### Health-related quality of life

The impact of Floor-HI on health-related quality of life was not statistically significant across any RAND-36 domains (*p* > 0.05) within the study period. The high baseline scores across domains suggest a ceiling effect, which is expected in healthy, community-dwelling middle-aged adults, thereby limiting the potential for detected improvement. Informal feedback from participants also suggested that certain RAND-36 items were perceived as linguistically ambiguous or culturally unfamiliar, which may have introduced response inconsistency and further limited the measure's sensitivity in this sample. This is consistent with observations by Wuttge and colleagues (50), who noted that the broader wording of RAND-36 items may introduce interpretive complexity compared to more domain-specific instruments such as Patient-Reported Outcomes Measurement Information System 29-item profile (PROMIS-29).

Moreover, the present findings must also be interpreted in the context of the small sample size. With only eight participants, it is difficult to draw definitive conclusions regarding the intervention's impact on health-related quality of life. Future studies with larger sample sizes may provide more definitive insights. Additionally, future studies should consider assessment of health-related quality of life through the PROMIS-29 as an alternative outcome measure. Compared to the RAND-36, PROMIS-29 employs more precise and contemporary language, has a shorter recall period, and may demonstrate greater sensitivity to subtle changes in high-functioning, community-dwelling samples, all of which are pertinent considerations for future trials of Floor-HI.

### Comparison with existing literature

These findings align with and extend existing literature on multicomponent fall prevention interventions that incorporate floor-rise training to enhance functional independence. The observed improvements in balance performance are consistent with systematic reviews demonstrating the efficacy of exercise-based interventions, particularly those targeting postural control through functional task training, including floor-rise activities (51, 52). Importantly, this study further contributes to the field by emphasizing floor familiarity and post-fall recovery skills, areas that remain underrepresented in conventional fall-prevention approaches.

The limited impact of Floor-HI on concern of falling is consistent with one recent floor transfer training study (53), but contrasts with findings from another floor transfer training (54) and exposure-based interventions that have demonstrated greater reductions in concern of falling (55–57). This may suggest that Floor-HI's exposure-based approach is more effective for enhancing balance capacity and task-specific confidence than for addressing psychological barriers associated with concern of falling. However, this interpretation should be considered preliminary and requires confirmation in larger, controlled studies.

### Limitations

Several limitations must be acknowledged in interpreting these findings. The study design did not include a control group, limiting causal inferences regarding the intervention's efficacy. While a multiple pre–multiple post design was used to assess outcome stability during the pre-intervention period (Weeks 0–3), this approach does not substitute for a controlled comparison condition. Moreover, the small sample size and homogeneous population (healthy, community-dwelling adults) restrict generalizability to broader populations at risk for falls. It should be noted that the extension of data collection following peer review also represents a potential limitation that should be considered when interpreting the findings.

Furthermore, the relatively short follow-up period (three weeks post-intervention) provides limited insight into the long-term sustainability of observed improvements. Likewise, the assessment schedule, while comprehensive, may have introduced practice or learning effects, which could partially account for some observed improvements. The use of printed surfaces to simulate hazardous environments, while novel, may not fully replicate the complexity of real-world fall situations. Additionally, the study's focus on middle-aged adults with low baseline concern of falling and high health-related quality of life may have limited the potential to detect meaningful changes in psychological and broader health-related outcomes.

## Conclusions

This exploratory study demonstrates that Floor-HI is feasible to deliver and acceptable to community-dwelling middle-aged adults, with a safety profile characterised by few mild and transient adverse events. Of the nine enrolled participants, eight completed the study (one withdrew following baseline assessment, prior to commencing the intervention) and reported high acceptability across multiple domains. Adverse events were mild and self-resolving throughout. Together, these findings support the potential for broader implementation and justify progression to controlled trials.

Moreover, functional outcomes showed significant improvements in balance, floor-rise ability, and turning capacity from pre- to post-intervention, with most improvements maintained at follow-up. However, the uncontrolled design prevents attribution of these changes to the intervention itself, as alternative explanations such as learning effects, regression to the mean, and natural variation cannot be excluded.

Floor-HI's unique dual-component approach, combining controlled exposure to floor environments with systematic training in post-fall recovery skills, addresses an important gap in current fall prevention approaches, which typically emphasize fall avoidance rather than fall management. The intervention's focus on building floor familiarity and developing post-fall recovery strategies represents a novel conceptual contribution that may complement existing prevention strategies. Overall, the encouraging feasibility, acceptability, and safety findings from this exploratory study support the need for controlled trials to establish efficacy and generalizability across diverse populations.

## Data Availability

The original contributions presented in the study are included in the article/Supplementary Material, further inquiries can be directed to the corresponding author.
