# Supplementary material for: Floor-Hugging intervention: findings from an exploratory study of a novel floor-exposure and post-fall contingency program
Source: Front Rehabil Sci. 2026 May 29;7:1730161. doi: 10.3389/fresc.2026.1730161 (PMC13260575; doi:10.3389/fresc.2026.1730161)
Supplement: Supplementary file 1 [file Supplementaryfile1.docx]

**Floor-Hugging intervention: findings from an exploratory study of a novel floor-exposure and post-fall contingency program**

Supplementary file

Table S1. Domain scoring for Mini-BESTest

| **Anticipatory** | |
| --- | --- |
| Week 0 | 6.0 (4.0-6.0) |
| Week 3 | 6.0 (4.0-6.0) |
| Week 6 | 6.0 (6.0-6.0) |
| Week 9 | 6.0 (6.0-6.0) |
| **Reactive postural control** | |
| Week 0 | 3.0 (2.0-6.0) |
| Week 3 | 3.0 (1.0-5.0) |
| Week 6 | 5.0 (3.0-6.0) |
| Week 9 | 5.0 (3.0-6.0) |
| **Sensory orientation** | |
| Week 0 | 6.0 (3.0-6.0) |
| Week 3 | 6.0 (5.0-6.0) |
| Week 6 | 6.0 (6.0-6.0) |
| Week 9 | 6.0 (6.0-6.0) |
| **Dynamic gait** | |
| Week 0 | 6.0 (5.0-8.0) |
| Week 3 | 7.0 (5.0-10.0) |
| Week 6 | 10.0 (10.0-10.0) |
| Week 9 | 10.0 (8.0-10.0) |

Table S2. Domain scoring for Sitting Rising test

| **Sittting down** | |
| --- | --- |
| Week 0 | 4.3 (1.5-4.5) |
| Week 3 | 4.0 (2.5-4.5) |
| Week 6 | 5.0 (3.5-5.0) |
| Week 9 | 5.0 (3.5-5.0) |
| **Getting up** | |
| Week 0 | 2.5 (1.5-4.5) |
| Week 3 | 3.8 (2.5-4.5) |
| Week 6 | 4.3 (3.0-5.0) |
| Week 9 | 4.0 (3.0-5.0) |
